# What are the costs of learning? Modest trade-offs and constitutive costs do not set the price of fast associative learning ability in a parasitoid wasp

**DOI:** 10.1007/s10071-019-01281-2

**Published:** 2019-06-20

**Authors:** Maartje Liefting, Jessica L. Rohmann, Cécile Le Lann, Jacintha Ellers

**Affiliations:** 1 Applied Zoology/Animal Ecology, Freie Universität Berlin, 12163 Berlin, Germany; 2 Animal Ecology, Vrije Universiteit Amsterdam, 1081 HV Amsterdam, The Netherlands; 3 Institute of Public Health, Charité-Universitätsmedizin Berlin, 10117 Berlin, Germany; 4 Center for Stroke Research Berlin, Charité-Universitätsmedizin Berlin, 10117 Berlin, Germany; 5 Université de Rennes, CNRS, ECOBIO (Ecosystèmes, Biodiversité, Evolution) UMR 6553, 263 Avenue du Général Leclerc, 35000 Rennes, France

**Keywords:** *Nasonia*, Global cost, Fecundity, Egg load, Memory, Relaxed selection

## Abstract

Learning ability has been associated with energetic costs that typically become apparent through trade-offs in a wide range of developmental, physiological, and life-history traits. Costs associated with learning ability can be either constitutive or induced, depending on whether they are always incurred or only when information is actively learned and memorized. Using lines of the parasitoid wasp *Nasonia vitripennis* that were selected for fast associative learning ability, we assessed a range of traits that have previously been identified as potential costs associated with learning. No difference in longevity, lipid reserves, tibia length, egg load, or fecundity was observed between the selected and control lines. All of these traits are considered to potentially lead to constitutive costs in the setup of this study. A gradual reversal to baseline learning after two forms of relaxed selection was indicative of a small constitutive cost of learning ability. We also tested for a trade-off with other memory types formed at later stages, but found no evidence that the mid-term memory that was selected for caused a decrease in performance of other memory types. In conclusion, we observe only one minor effect of a constitutive cost and none of the other costs and trade-offs that are reported in the literature to be of significant value in this case. We, therefore, argue for better inclusion of ecological and economic costs in studies on costs and benefits of learning ability.

**Electronic supplementary material:**

The online version of this article (10.1007/s10071-019-01281-2) contains supplementary material, which is available to authorized users.

## Introduction

Environmental variation is a ubiquitous feature of life, and nearly all organisms experience variation in essential conditions such as temperature and food availability on a daily, seasonal, or lifetime scale. Phenotypic plasticity in physiology and behaviour is one way to deal with temporal and spatial variation on varying timescales. When environments vary within the lifetime of an individual, there is selection for flexible and reversible phenotypic plasticity to avoid costly mismatches (de Jong 1995; Snell-Rood 2013). However, the level of plasticity in traits has been found to vary between populations (Liefting and Ellers 2008), which implies that there is a cost involved in being able to respond plastically. To understand the evolution of phenotypic plasticity of traits, we need to understand the benefits and costs involved (DeWitt et al. 1998; Callahan et al. 2008).

The ability to learn is often considered a special type of phenotypic plasticity (Kawecki 2010; Mery and Burns 2010). Learning is the process of integrating information from previous experiences about the environment into memory, which allows for behavioural adjustments later in life. This makes learning potentially one of the fastest ways to reversibly modify phenotype in response to changing environmental conditions (Raine 2009), such as human-induced rapid environmental change (Sih 2013). Just like other forms of behavioural and phenotypic plasticity, learning is expected to be beneficial when environmental conditions are variable but to some extent predictable within the lifetime of an individual (Stephens 1991; Ernande and Dieckmann 2004; Dunlap and Stephens 2016). Several recent empirical studies indeed support these theoretical predictions (Mery and Kawecki 2002, 2004; Dunlap and Stephens 2009).

Cognitive abilities of species result from a balance of costs and benefits of learning (Johnston 1982). Traditionally, costs of learning are considered to be either constitutive or induced. Induced (or operating) costs are the costs associated when information is actively learned, memorized and recalled, and these costs are often of an energetic nature (Johnston 1982; Mery and Kawecki 2005; Burns et al. 2011; Jaumann et al. 2013). On the other hand, simply having the ability to learn may require energetically costly neural networks laid down and maintained, whether these are used or not. These costs are referred to as constitutive (or global) costs, they are paid even when the ability to learn is not used (Mery and Kawecki 2003; Snell-Rood et al. 2009, 2011). Costs of suboptimal behaviour during learning or of learning misguided information are of a different order and are usually referred to as ecological or economic costs (Dunlap and Stephens 2016). Where ecological and opportunity costs are usually approached theoretically in modelling studies, induced and constitutive costs have been primarily addressed empirically in controlled environments through trade-offs with a wide range of developmental, physiological, and life-history traits (Dunlap et al. 2019), but see Eliassen et al. (2009, 2017) and Evans and Raine (2014).

Such trade-offs occur, because there are relatively high energetic costs involved in building and maintaining the synaptic connections underlying the integration, storage, and retrieval of information (Dukas 1999; Laughlin 2001; Niven and Laughlin 2008). For example, having relatively large brains was found to be associated not only with higher learning performance, but also with reduced gut tissues (Kotrschal et al. 2013) and fecundity (Snell-Rood et al. 2011; Kotrschal et al. 2013). Trade-offs with learning performance have also been found for delayed juvenile development (Christiansen et al. 2016), decreased longevity (Burger et al. 2008; Lagasse et al. 2012), reduced larval competitive ability (Mery and Kawecki 2003), and reduced foraging careers (Evans et al. 2017). The high energetic costs of learning can cause cognitive impairment in stressed honeybees (Jaumann et al. 2013). Similarly, long-term memory formation reduces resistance to food and water stress in flies (Mery and Kawecki 2005) and a possible consequence is that this type of memory is disabled under conditions of food deprivation (Plaçais and Preat 2013). In addition, a trade-off between a form of middle-term memory and long-term memory has been described in *Drosophila,* where selection for one memory phase resulted in reduced performance in the other memory phase (Lagasse et al. 2012). From these studies, the costs of learning seem apparent from many different empirical studies, but the results are divergent. It tells us that costs can be paid in many different ways, but not whether some trade-offs are more likely to occur than others. An integrative approach that moves beyond the mere description of costs found, but also includes observations of the absence of expected costs and trade-offs would be insightful in this matter.

Artificially selected lines provide an excellent opportunity to explore above-mentioned costs in an integrative manner, because they can easily be compared to control lines that experienced similar treatment and originated from the same genetic background (Mery and Kawecki 2002). In this study, we set out to quantify a series of potential costs involved in fast associative learning ability, using lines of the parasitoid wasp *Nasonia vitripennis* that had been selected to rapidly form learned associations. Artificial selection on standing genetic variation in cognitive abilities resulted in selected lines that readily formed appetitive associations within 10 generations of selection (Liefting et al. 2018). We subsequently measured these selected and control lines for multiple traits that have been associated with constitutive costs in the literature. One potential cost is already described in the previous work on these lines; brain size and relative size of different neuropils did not differ between wasps of the selected and control lines (Liefting et al. 2018).

To test for any constitutive costs associated with fast learning ability, we quantified several life-history traits in the absence of a learning experience. These traits included longevity, lipid reserves, tibia size, egg load, and realized fecundity. Females will start searching for hosts directly after emergence. Any reduction in longevity would be ecologically relevant as this reduces the time available to find a concealed host and reproduce. Lipid content and tibia length are suitable and established proxies for female fitness in parasitoid wasps and even small trade-offs in such traits are, therefore, highly relevant. *Nasonia vitripennis* females emerge with no mature eggs (Jervis and Ferns 2004), but with reserves of lipids and glycogen that they can use for developing eggs and sustenance while searching for fresh hosts (Rivero and West 2002). The fact that female body size is correlated with the number of eggs (Rivero and West 2002) makes tibia length also a good proxy for fitness. Females start to produce mature eggs after emergence and, therefore, egg load at 24 h and realized fecundity as measured by the number of offspring emerging from a host are both well-established ways of estimating effects on fitness directly.

A second method to assess constitutive costs of learning ability was to determine whether the improved learning ability of the selected lines would revert back to the baseline learning ability of the control lines when the source of selection was either weakened or completely removed. Both situations are forms of ‘relaxed selection’ (Lahti et al. 2009). Finally, we also tested for trade-offs between memory types by testing memory retention over several days after a single conditioning trial in both the selected and control lines. As wasps of the selected lines were chosen for their improved learning ability 24 h after conditioning, the resulting adjustments in learned behaviour are most likely the result of a combination of high learning rate and quick formation of middle-term memory (Liefting et al. 2018). If the ability to form strong middle-term memories trades off with long-term memory (as described in Lagasse et al. 2012), this would be a strong indication of a cost, either induced or constitutive or a combination of both, being at play.

## Materials and methods

### Study system and selected lines

All experiments were done with *N. vitripennis* (Chalcidoidea: Pteromalidae), a gregarious parasitoid wasp that lays eggs in dipteran pupae (Whiting 1967) and is able to form learned associations with odours and colours (Oliai and King 2000; Hoedjes et al. 2012). We used control and selected lines from a previous experiment in which a genetically variable *N. vitripennis* population was subjected to artificial selection for improved associative learning (Liefting et al. 2018). In short, eight random groups of wasps were set up from a laboratory culture (van de Zande et al. 2014). We allocated four of these to a selection regime, and females for the next generation were selected based on prompt associative learning (‘selected lines’). The other four random groups were allocated to a control regime and females for the next generation were chosen at random, independent of their learning performance (‘control lines’) (Liefting et al. 2018).

Wasps were conditioned on either the colour blue or yellow by allowing small groups of females to probe and feed off host pupae for 1 h in petri dishes placed on coloured paper. After this conditioning phase, the females were briefly placed on the other colour without hosts present to familiarize them with this non-rewarding colour. After 24 h, conditioned females were released in groups of 10 in a T-maze with the two colours on either side and their distribution was determined after 10 min. Memory retention of the learned colour preference was quantified with a performance index (PI). A PI is calculated based on the sum of the percentages of correct choices of the 10 wasps in the T-maze minus 50% (i.e., the expected percentage in the absence of learned associations), as measured for both colours simultaneously. Hence, the PI value can range from − 100 (perfect avoidance) to 100 (perfect preference) with a value of 0 indicating no learned associations. The ability to rapidly form a learned association substantially increased from a PI of 23 (SE ± 5.2) to 34 (SE ± 8.2) after only 10 generations of selection. For further details on the conditioning assay, selection regime and PI, see Liefting et al. (2018). The wasps were reared at 25 °C during the experiments under a 16 h light/8 h dark regime; only the behavioural essays were performed at 20 °C.

### Post-selection measurements

**Fig. 1:**
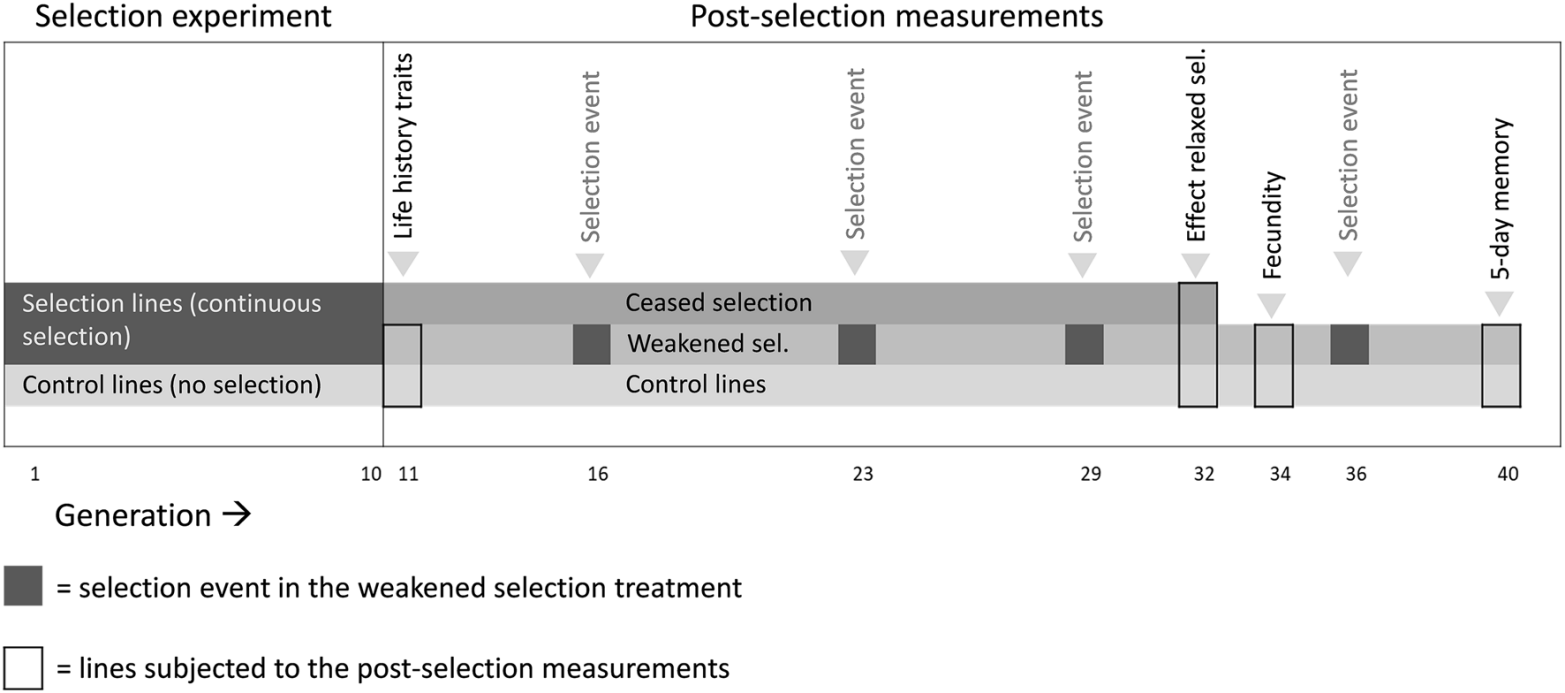
Schematic overview of the selection experiment (generations 1–10) and the post-selection measurements (generations 11–40). Several life-history traits were investigated in the selected and control lines at generation 11. Relaxed selection after 10 generations of selection was realized by either weakening selection to a selection event at generation 16, 23, 29 and 36 (weakened selection) or by complete cessation of selection (ceased selection). Effects of these two relaxed selection treatments were estimated at generation 32. At generation 34, fecundity was measured in the selected and control lines, and at generation 40, the 5-day memory dynamics of the selected and control lines were recorded

After 10 generations of continuous selection, the four selected and four control lines were assessed for several life-history traits and aspects of learning ability. These life-history traits were longevity, lipid content, tibia size, and egg load at 24 h and were measured in females of generation 11. After 10 generations of continuous selection, the selection lines were exposed to two different regimes of relaxed selection (Lahti et al. 2009). Monitoring whether a phenotypic trait gradually or rapidly reverts back to the original state can reveal insights about possible costs involved in maintaining this phenotype (Brakefield 2003) while also eliminating any parental effects. In one treatment, selection on associative learning ability was halted for all lines (‘ceased selection’). In the other treatment group, the selected lines were subjected to three subsequent selection events with one every 6–7th generation (‘weakened selection’). The control lines were also maintained an additional 22 generations under the exact same conditions as before. At generation 32, the learning ability of the lines in all three treatments was compared. In addition to egg load (number of mature eggs in ovaries of 24-h-old females), realized fecundity of the selected and control lines was also measured in generation 34. At generation 40, after establishing that the selected lines still differed in learning ability from the control lines in generation 36, the pattern in memory retention over 5 days following a single conditioning was measured for all lines. Figure 1 provides a schematic overview of which measurements were performed at which generation.

### Constitutive costs; life-history traits

To estimate the average longevity of wasps of the selected and control lines, 40 mated females of each of the four selected and four control lines of generation 11 were collected on the day of emergence. Each female was kept individually in a glass vial at 25 °C and provided with a small cotton ball soaked in water and a drop of diluted honey on the plug closing the vial. Water and honey were replenished every 2 days. The survival of each individual wasp was checked daily until death. Only females that did not survive the 1st day were replaced with a female of the same age. The longevity experiment ended after the final surviving wasp died after 62 days.

Lipid content of females was determined based on the method by David et al. (1975) that applies well to parasitoids (Visser et al. 2010). A total of 10 mated females per line per treatment were collected on the day of emergence and frozen separately. Whole wasps were subsequently freeze-dried for 2 days, after which dry weight was determined and individuals were immersed in 4 ml of ether in a glass vial. After 24 h, the ether was removed and insects were dipped in fresh ether and then left in a fume hood for at least 4 h until the remaining ether evaporated. The wasps were then freeze-dried again for 2 days to determine dry weight after ether extraction. Lipid content was calculated as the difference in weight before and after ether extraction. Tibia length of the right hind leg, a standard measurement of size in parasitoids (Godfray 1994), was measured of 10 females per line per treatment using a dissecting microscope and an ocular micrometre.

To determine egg load, the number of mature eggs in the ovaries of 1-day-old females of selected and control lines was counted. A total of 10 females were dissected under a dissecting microscope, the ovaries placed on a slide and the number of mature eggs determined (following Edwards 1954). As an additional measure to egg load, realized fecundity of 10 non-fed, 1 day-old females of each of the four selected and four control lines of generation 34 was measured by letting these females oviposit in a (weighed) host pupa (*Calliphora vomitoria*) for 15 h. Females will sting the host, feed on host liquids and lay eggs on the outside of the host underneath the puparium. The host meal will also stimulate egg production. After 15 h, the females were transferred to a fresh host pupa to continue egg laying until they died (approximately 2–3 days later). The maximum number of wasps that can complete development on one host varies with the size of the host and, therefore, with the host species (Whiting 1967). The number of wasps emerging from a large host species such as *C. vomitoria* ranges from 30 to 50 wasps (van der Merwe 1943; Rivers and Losinger 2014). Females can lay more eggs, but will usually not exceed this number unless under strong competition, because this results in smaller and less viable offspring. After approximately 2 weeks at 25 °C, the number of emerging offspring was counted for each host, and the sex ratio of the progeny measured (i.e., number of emerged males on the total number of emerged individuals).

### Constitutive costs: relaxed selection

At generation 32, the PI was measured for the selected group with ceased selection, the selected group with weakened selection and the control lines. Unfortunately, one of the replicates of the ceased selection lines had to be discarded, because the number of viable wasps declined dramatically due to an unidentified cause. Therefore, only three lines per treatment (three control lines, three ceased selection lines, and three weakened selection lines) were compared by excluding the control line that was paired to the lost line in the ceased selection treatment. The wasps were conditioned during a single conditioning trial and memory retention after 24 h was measured in the T-maze. A PI is calculated based on the sum of the percentages of correct choices of the 10 wasps in the T-maze minus 50% (i.e., the expected percentage in the absence of learned associations), as measured for both colours simultaneously. Therefore, one PI is based on the behaviour of 20 wasps. A total of 12 PIs was established per line per treatment (three control lines, three ceased selection lines, and three weakened selection lines).

### Trade-offs between memory phases

To test whether the enhanced 24 h memory of the selected lines trades off with another memory type, the PI of 2-day-old females of generation 40 was measured on days 1–5 after conditioning, and compared between the four selected (weakened selection) and four control lines. A PI is calculated based on the percentage of correct choices of the 10 wasps in the T-maze minus 50% (i.e., the expected percentage of wasps choosing correctly in the absence of learned associations), as measured for both colours simultaneously (i.e., 20 wasps in total for one PI). A total of 10 PIs per line and per treatment on each of the 5 days was established.

### Statistical analyses

To analyse the longevity data, we first estimated Kaplan–Meier survival curves for each group (pooled selected and pooled controls). Survival time was measured in days from the time of experiment start until the time of death or loss. Any deviations from the proportional hazards assumption were graphically checked by plotting scaled Schoenfeld residuals versus time. Overall survival was then compared using the log-rank test. Wasps that went missing during the experiment (*n* = 4) were recorded as such and censored in the analyses at time of loss. The time of death cannot be established, but the information up until the moment they went missing is still relevant. A mixed-effects cox regression model was then used to compare survival between selected and control groups with line as a random factor using the package ‘coxme’ in R (Therneau 2018). We present a hazard ratio with corresponding 95% confidence interval.

Differences in lipid content of selected and control lines were assessed with a linear mixed model with log-transformed lipid content as the dependent variable, log-transformed weight after ether extraction as a covariable, and line as a random factor. Effects on tibia length were analysed with a linear mixed model with treatment (selected or control) as a fixed factor and line as a random factor. The function ‘lmer’ in the ‘lme4’ package (Bates et al. 2015) was used to fit the linear mixed models.

Effects on egg load at 24 h were estimated with a zero-inflated generalized linear mixed model using a negative binomial distribution in the package ‘glmmTMB’ (Brooks et al. 2017). In the model, number of eggs was the dependent variable, treatment (selected or control) was included as a fixed factor and line as a random factor. Number of offspring emerging from the first and second host was also analysed with a zero-inflated generalized linear mixed model using a negative binomial distribution. The model included treatment (selected or control) and weight of the host as fixed factors and line as a random factor. The sex ratio of the emerging offspring per host was analysed with a linear mixed model with the sex ratio as the dependent variable, treatment and weight of the host as fixed factors, and line as a random factor.

In the analysis of the relaxed selection experiment, the PIs of the three different treatment groups (ceased selection, weakened selection, and control) were analysed with a linear mixed model with PI as the dependent variable, treatment as a fixed factor, and line as a random factor. Differences between the treatment groups were analysed by pairwise comparisons based on the estimated marginal means using the package ‘emmeans’ (Lenth et al. 2018). In the analysis of possible trade-offs between memory phases, effects on memory dynamics of selected and control lines over 5 days after a single conditioning trial were estimated with a linear mixed model with PI as the dependent variable, treatment as a fixed factor, and day after conditioning as a fixed factor, including a treatment*day interaction and line as a random factor. Differences in PI between selected and control lines were analysed per day in a post hoc analysis.

All analyses were performed in R 3.4.4 (R Core Team 2019). For all linear (mixed) model analyses, assumptions for homoscedasticity and normality were met based on visual inspection of the distributions of predicted and residual values.

## Results

### Longevity

**Fig. 2:**
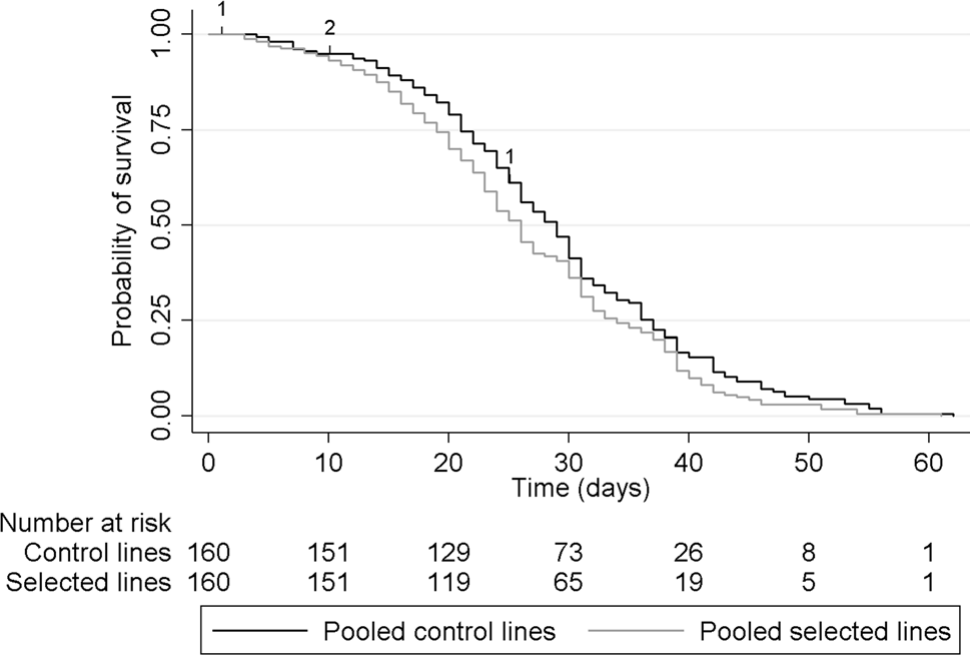
Kaplan–Meier survival curves show the survival probabilities over time for the selected (*N* = 160) versus control (*N* = 160) lines. The last wasp died after 62 days. The experiment started out with 40 females per line per replicate treatment line and 4 females were lost to follow-up (time points of censoring are indicated with small numbers on the graphs)

The Kaplan–Meier survival curve of the pooled selected lines lies consistently below the survival curve of the pooled control lines (Fig. 2), but the treatment groups did not differ significantly in their survival in this crude comparison (*N* = 160 per group, four lost to follow-up, Log-rank statistic = 3.7, *p* = 0.054). No violations of the proportional hazards assumption were observed. The hazard ratio of death for the selected group compared with the control group was found to be 1.22 (95% CI 0.88–1.69) in the mixed-effects Cox regression model. Survival curves for the individual lines are shown in Figure S1 in supplementary material.

### Lipid content and tibia length

**Fig. 3:**
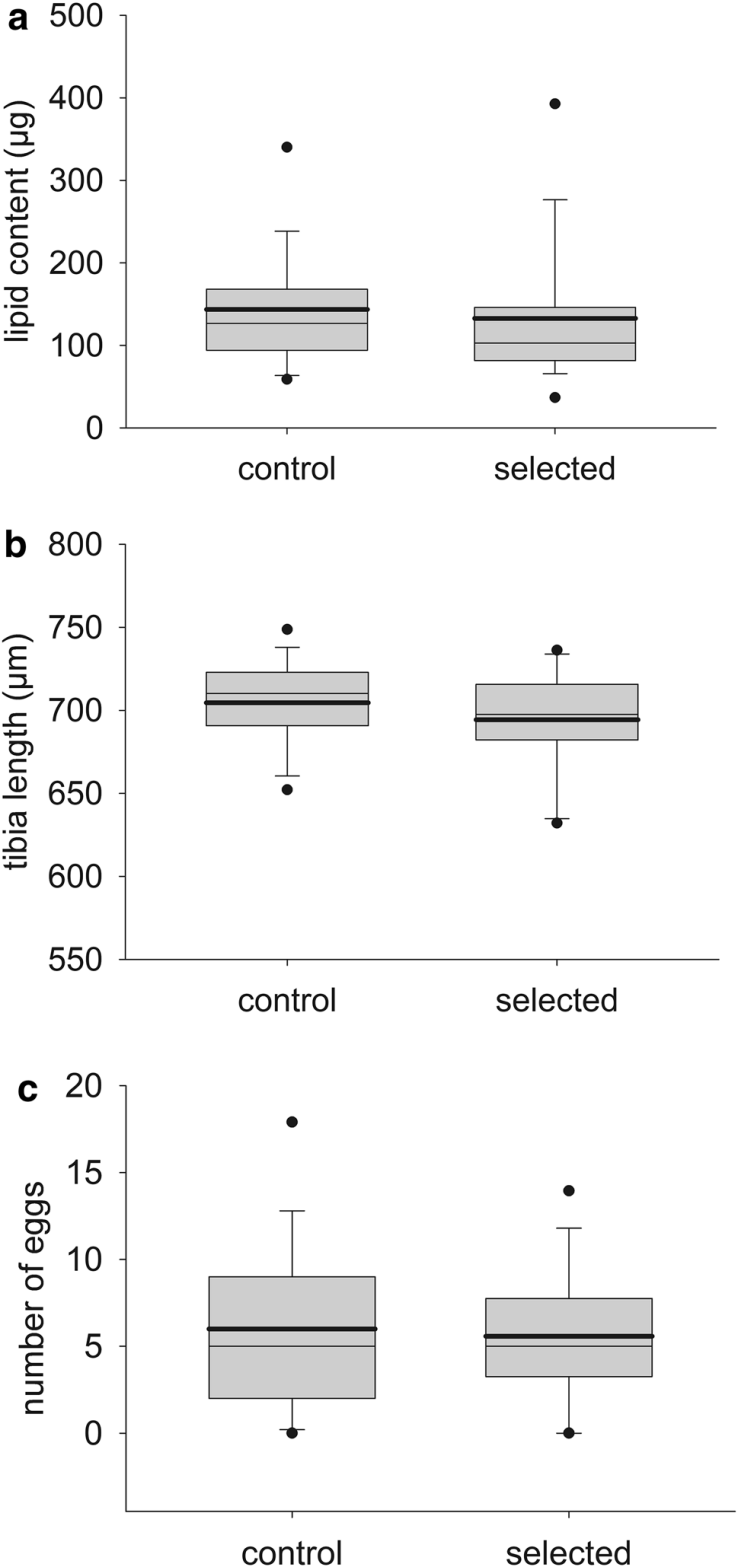
Box plots with the results for **a** lipid content, **b** length of the right hind leg tibia, and **c** number of mature eggs in the ovaries of 24-h-old females of the four control and the four selected lines as measured in generation 11 after ending the selection experiment at generation 10. Measurements were on 10 females per line per treatment. The box indicates the 25th and 75th percentiles, the whiskers indicate the 10th and 90th percentiles, and the points the 5th and 95th percentiles. A thin line within the box marks the median, the bold line the mean. One extreme outlier in the tibia data (not shown) was trimmed from the data set, see main text

There was a significant effect of body weight on lipid content of *N. vitripennis* females (dry weight after ether extraction: *F*_1, 75_ = 14.3, *p* = 0.0003), but there was no difference in lipid content between the selected and control lines (effect treatment *F*_1, 74_ = 1.0, *p* = 0.32; Fig. 3a). There was also no difference found in tibia length between selected and control lines (*F*_1, 74_ = 2.5, *p* = 0.12; Fig. 3b). One extreme outlier with a 3.75 standard deviation below the mean was treated as a missing value (not shown in Fig. 3b). We ran the same model while including the outlier in a sensitivity analysis and this did not change the conclusion. However, treating the outlier as a missing value drastically improved the model fit.

### Egg load and realized fecundity

There was no effect of treatment (selected or control) on egg load in the conditional model after correcting for the zero inflation (cond. model *z* value = 0.08, *p* = 0.94; Fig. 3c), or in the frequency of absence of eggs (zero-inflated model *z* value = 0.4, *p* = 0.67). Likewise, the zero-inflated mixed model estimated no difference in realized fecundity between selected and control lines. The number of offspring emerging from the first host did not differ between treatments (zero-inflated model *z* value = 1.5, *p* = 0.13, cond. model *z* value = 1.6, *p* = 0.10; Fig. S2), nor did the number of offspring from the second host (zero-inflated model *z* value = 0.6, *p* = 0.53, cond. model *z* value = 0.9, *p* = 0.38). The sex ratio of the emerging offspring was also similar for selected and control lines in both the first host (*F*_1, 57_ = 1.0, *p* = 0.33) and the second host (*F*_1, 58_ = 0.4, *p* = 0.52), i.e., varying between 0.03 and 0.45 proportion males of the total offspring.

### Relaxed selection treatments

**Fig. 4:**
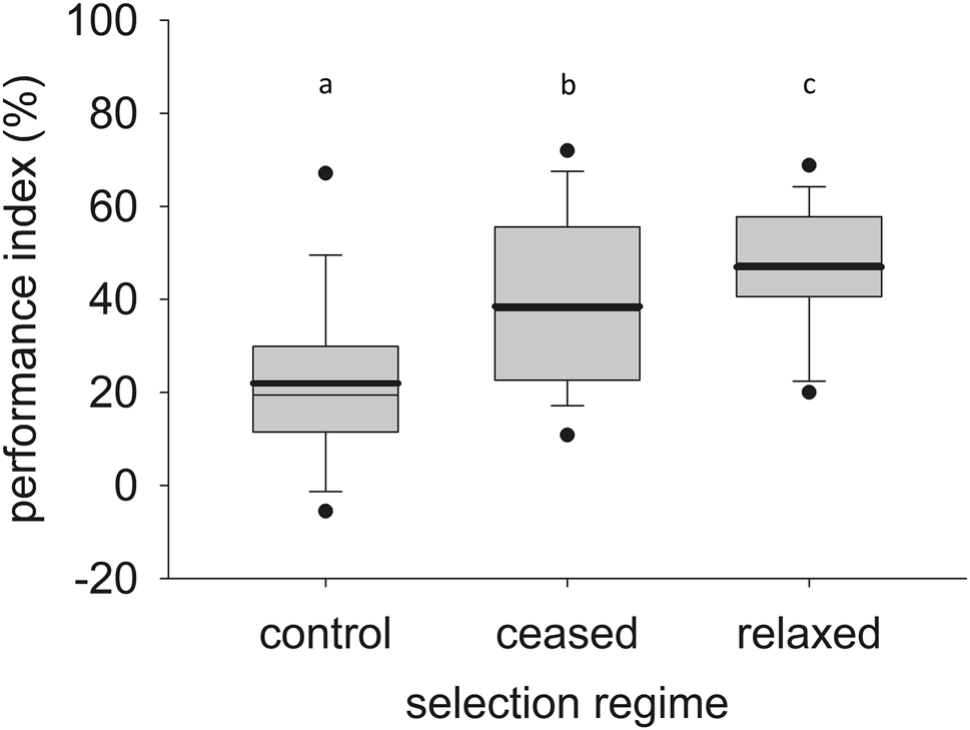
Box plot showing the performance index (PI) measured at generation 32 of lines that have been subjected to three different selection regimes since the end of the selection experiment; the control lines that have never been subjected to selection, and the lines that have been subjected to either one of two forms of relaxed selection after the 10th generation of selection; either weakened selection with a selection event every 6–7 generations or all selection activity ceased. A total of 12 PIs was measured in each of the three lines of each treatment. The box indicates the 25th and 75th percentiles, the whiskers indicate the 10th and 90th percentiles, and the points the 5th and 95th percentiles. A thin line within the box marks the median, the bold line the mean

During the selection experiment, there was substantial variation in PI values among generations, so that only a comparison within the same generation is meaningful. Therefore, we only report the differences in PI values amongst the 3 relaxed selection treatments in generation 32, and not compare them to the PI values at generation 10, which are roughly in the same range. A strong effect of relaxed selection treatment (selected or control) on PI was found (*F*_2, 103_ = 19.4, *p* < 0.0001), with the lowest PI in wasps from the control treatment and the highest PI in wasps from the weakened selection treatment (Fig. 4). The PI differed significantly among all three treatments (pairwise analysis of contrasts: ceased versus control (Est. = 16.6, S.E. = 4.1, *p* = 0.0001), ceased versus weakened (Est. = − 8.5, S.E. = 4.1, *p* = 0.04), and control versus weakened (Est. = − 25.0, S.E. = 4.1, *p* < 0.0001)).

### 5-day memory pattern

**Fig. 5:**
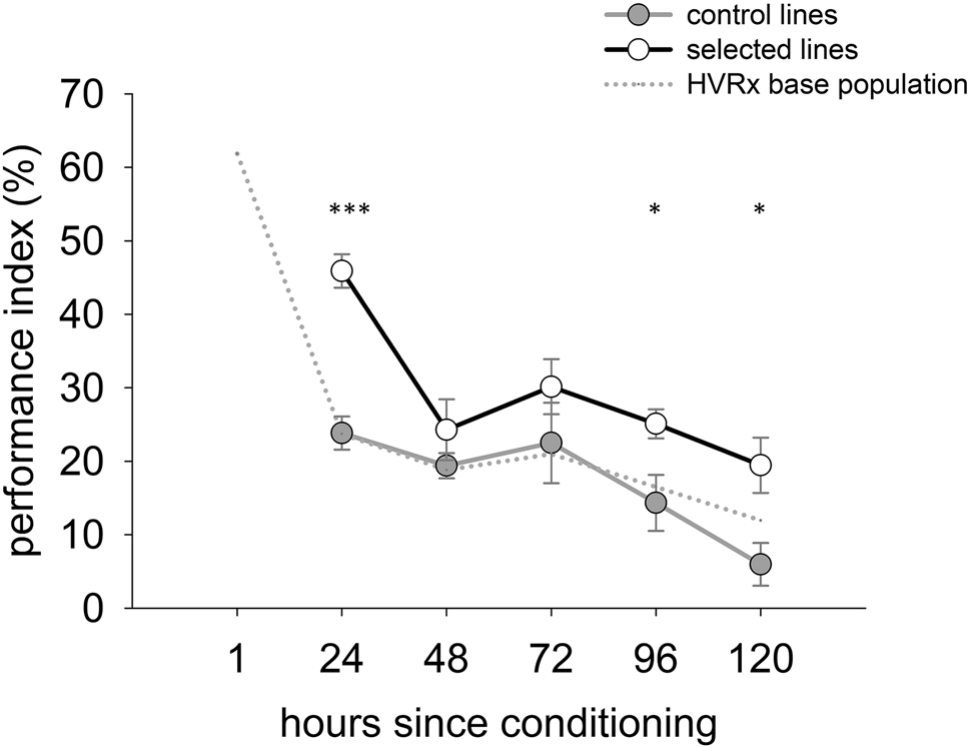
Performance index (PI) as a measure of learned preferences for the control and selected lines of generation 40, measured on days 1–5 after a single conditioning trial. For reference, the 5-day memory dynamics of the base population from which the control and selected lines were drawn (HVRx, see van de Zande et al. 2014) is included as a grey dotted line (no SE bars are included to improve visual discrimination). On each day, 10 PIs were measured per line per treatment. Statistically significant differences in PI per day between the selected and control lines are indicated with asterisks (*p < 0.05, ***p < 0.001)

The memory dynamics over a 5-day period after a single conditioning trial showed a strong effect of time after the conditioning (*F*_1, 393_ = 32.9, *p* < 0.0001) and of treatment (selected or control) (*F*_1, 393_ = 26.4, *p* < 0.0001) on the level of the PI. However, there was no interaction observed between these factors (*F*_1, 393_ = 0.5, *p* = 0.49), indicating that the pattern in memory decline is the same for selected and control lines. Based on a post hoc analysis, the PI was higher for the selected than the control lines on days 1, 4, and 5 as marked in Fig. 5 (statistical output in supplements).

## Discussion

To identify trade-offs and costs involved in learning, we measured life-history and other traits in lines selected for rapid associative learning ability and their control lines. No differences in longevity were found between selected and control lines under accommodating controlled conditions. There were also no constitutive costs detectable through trade-offs with lipid content, tibia length, egg load, fecundity, or memory phases. The only effect we found was a gradual reversal to baseline learning ability when the selective pressure is removed, which is indicative of a small constitutive cost of the high learning ability. When selection for learning ability was weakened or ceased completely, the lines in the ceased selection treatments showed a stronger decline in PI than the lines in the weakened selection treatment. These results show that the high learning ability observed in the selected lines is not entirely without constitutive costs, but that these costs were not found to be as pronounced as reported in other studies (Burger et al. 2008; Burns et al. 2011; Snell-Rood et al. 2011; Lagasse et al. 2012).

Neither lipid content nor tibia length differed between wasps from the selected and control lines (Fig. 3a, b), so there is no a priori (dis)advantage between females from either treatment. These traits serve as proxies for fitness, but also when measured directly no difference was observed in egg load at 24 h after emergence or realized fecundity between selected and control lines. As mentioned earlier, *N. vitripennis* females emerge without mature eggs (Jervis and Ferns 2004). They will start producing mature eggs after emergence, resulting in about 5–15 mature eggs developed after 24 h, with egg production starting to decline after day 2 due to reabsorption of eggs in the absence of a host meal (Edwards 1954). A trade-off between fast learning ability and reproductive investment resulting in a lower egg load 24 h after emergence could potentially affect fitness in a competitive environment. However, females from selected and control lines had a similar number of mature eggs in their ovaries (Fig. 3c) and their realized fecundity, measured as the number of offspring emerging from the first or the second host, was also similar (Fig. S2 in supplementary material). The number of offspring emerging from the hosts never exceeded 50, as predicted from the literature. The number of eggs laid in the first host was higher than the number laid in the second host, and the females started dying after parasitizing in the second host. Therefore, availability of fresh hosts does not seem to have been limiting. Even though there is evidence in the literature for trade-offs between (enhanced) learning and fecundity or reproduction ability (e.g., Snell-Rood et al. 2011; Kotrschal et al. 2013), such differences could not be confirmed in this study based on direct and indirect measurements of fecundity.

Reducing or completely removing selection for improved learning led to a difference in PI between the three post-selection treatments (Fig. 4). Monitoring changes in phenotype during period(s) of relaxed selection can both eliminate parental effects and reveal insights about fitness relationships relevant to the trait under selection (Brakefield 2003). If costs are high, a rapid decline in frequency of the extreme learning phenotypes is expected (Beldade et al. 2002; Brakefield 2003). When the artificial selective pressure was completely removed (ceased selection), the average value of PI was reduced compared to the PI of the lines under weakened selection, although the PI in both treatments remained higher than the PI of the control treatment. We conclude that when selection is completely ceased the learning ability slowly reverts back to the baseline learning ability of the control lines, but that weakened selection (a selection event every 6–7th generation) appears to be sufficient to halt or further slow down that process. This is not unexpected as artificial selection regimes often change allele frequencies of standing genetic variation rather than lead to fixation of loci (Teotónio et al. 2009). Variation in learning ability is likely not caused by a few ‘learning genes’ with large effects on learning ability, but rather depend on more subtle variation in a large number of genes associated with underlying neural pathways or processes (Barrick and Lenski 2013), as was also concluded in a genomic study of the selected lines used in this study (Kraaijeveld et al. 2018). The reversal towards baseline learning ability in lines that no longer experience selection could suggest that a certain constitutive cost is involved, although this cost is likely to be small considering the gradual pace of the decline (Hoffmann et al. 2001; Raymond et al. 2001; Beldade et al. 2002).

Different memory types can be formed in *N. vitripennis*, depending on the type of experience and the duration of this experience (Schurmann et al. 2012, 2015). Directly after an experience, anaesthesia-sensitive/short-term memory (STM) is formed, which can be followed by two forms of intermediate memory (MTM) and ultimately consolidated into a long-term memory type (LTM) that requires protein synthesis. In the conditioning assay of our selection experiment, females were allowed to drill into and feed off hosts for 1 h, which is sufficient to lead to LTM according to Schurmann et al. (2012). However, the selection for an improved learned association took place 24 h after conditioning, which is during the formation of the intermediate transient memory type (MTM) preceding LTM. Although different memory types can co-exist without interference (Margulies et al. 2005; Schurmann et al. 2012), there is also evidence of a possible trade-off between memory types (Lagasse et al. 2012). If energetic costs are limiting memory formation, this could result in a trade-off between memory types. The selected lines may thus have possibly formed improved MTM at the cost of another memory type like LTM. Nevertheless, the results of the 5-day memory pattern of the selected and control lines show no sign of a trade-off between memory types (Fig. 5).

The decline to 48 h memory is sharper in the selected lines than might have been expected from the overall pattern (Fig. 5). Similar temporal depressions in memory retention or ‘dips’ have been described in other species from chickens (Gibbs and Summers 2002) to honeybees (Eisenhardt 2006) and typically appear at predicted phase transitions (DeZazzo and Tully 1995). In *Nasonia*, a transition from an anaesthesia-resistant memory (ARM or MTM I) to MTM II is predicted to occur around this time based on treatment with anaesthetics (Schurmann et al. 2012, 2015). The fact that the score for memory drops sharply after 24 h memory could be an indication that in particular the ARM (or MTM I type) is more strongly consolidated in the selected lines, leading to a stronger response in LTM as well (hence also the significantly different response in PI on days 4 and 5), but that this ARM does not automatically lead to stronger consolidated MTM of the second type (typically ranging from days 1 to 3). Whether the ARM and LTM are coupled or not remains unclear; the higher response on days 4 and 5 in the selected lines could also be caused by a lag effect of memory degradation.

Overall, this study did not provide evidence for costs of fast associative learning affecting longevity, fecundity, body size, and other types of memory, even though the reversal towards baseline learning ability when the selection pressure was removed suggests that some constitutive costs are involved. A previous study on the same selection experiment also reported a lack of difference between the selected and control lines for brain size and brain sections (Liefting et al. 2018). Although costs of learning reported in the literature are not evident in our results, the list of possible constitutive and induced costs is much larger than the ones explored here and it is likely that we have not yet defined the most relevant learning-related cost for this species. We would, therefore, echo recent arguments for a better inclusion of opportunity and economic costs in the study of cognitive evolution (Dunlap and Stephens 2016). The economic costs of learning go beyond the expenses of building and maintaining the learning machinery (Eliassen et al. 2009, 2017). Taking the time to learn something and establish new behaviours comes at a cost, as well as the presumably poor performance during that time. There could also be costs involved in learning information very quickly if this information turns out to be unreliable, something that is particularly relevant to short-lived insects (Evans and Raine 2014; Evans et al. 2017; de Bruijn et al. 2018; Vosteen et al. 2019). There are reasons to think that these costs are at least as important as the mechanistic costs of learning (Stephens 1991; DeWitt et al. 1998). Any ecological costs in addition to the modest constitutive costs as we have described in this study might set the real ‘price’ for fast associative learning.

## Electronic supplementary material

Below is the link to the electronic supplementary material.

Supplementary material 1 (DOCX 727 kb)
