# Supplementary material for: What are the costs of learning? Modest trade-offs and constitutive costs do not set the price of fast associative learning ability in a parasitoid wasp
Source: Anim Cogn. 2019 Jun 20;22(5):851–61. doi: 10.1007/s10071-019-01281-2 (PMC6687694; doi:10.1007/s10071-019-01281-2)
Supplement: Supplementary file 1 — Supplementary material 1 (DOCX 727 kb) [file 10071_2019_1281_MOESM1_ESM.docx]

**Supplementary material with manuscript “What are the costs of learning? - Modest trade-offs and constitutive costs do not set the price of fast associative learning ability in a parasitoid wasp”**

Liefting, M., J. L. Rohmann, C. Le Lann, J. Ellers

**
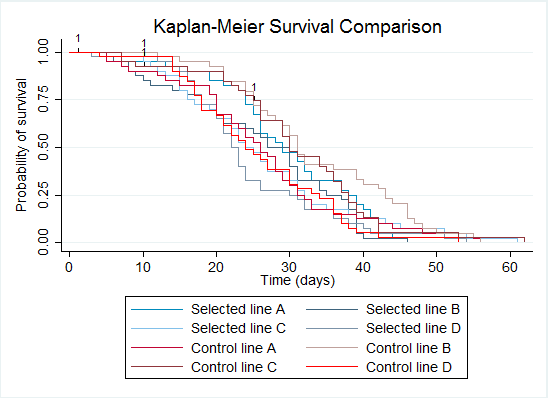
**

**Fig. S1** The Kaplan-Meier survival curves show the survival probabilities over time for the 4 selected (N=40) versus 4 control (N=40) lines. The last wasp died after 62 days. The experiment started out with 40 females per line per replicate treatment line and 4 females were lost to follow-up (time points of censoring are indicated with small numbers on the graphs).


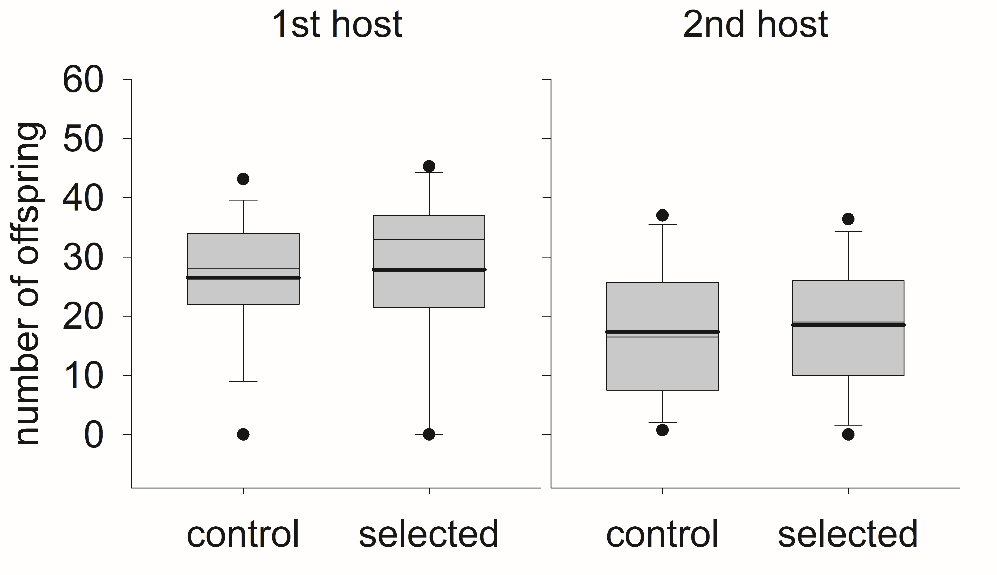


**Fig. S2** Box plot showing the total number of offspring emerging per parasitized host. Mated females (24 hrs old) of the selected and control lines of generation 34 were provided with a host for 15 hours to parasitize. After 15 hrs they were transferred to a second host to lay a second batch of eggs and remained there until they died. The sex ratios did not differ between the control and selected lines. Measurements were conducted on 10 females per line per treatment. The box indicates the 25^th^ and 75^th^ percentiles, the whiskers indicate the 10^th^ and 90^th^ percentiles, and the points the 5^th^ and 95^th^ percentiles. A thin line within the box marks the median, the bold line the mean.

**Statistical output 5-day memory; differences in PI per day**

DAY 1

day1nul: PI1 ~ (1 | line)

day1: PI1 ~ treatment + (1 | line)

Df AIC BIC logLik deviance Chisq Chi Df Pr(>Chisq)

day1nul 3 741.52 748.67 -367.76 735.52

day1 4 724.50 734.02 -358.25 716.50 19.023 1 1.291e-05 ***

---

DAY 2

day2nul: PI2 ~ (1 | line)

day2: PI2 ~ treatment + (1 | line)

Df AIC BIC logLik deviance Chisq Chi Df Pr(>Chisq)

day2nul 3 729.75 736.89 -361.87 723.75

day2 4 730.78 740.31 -361.39 722.78 0.9672 1 0.3254

---

DAY 3

day3nul: PI3 ~ (1 | line)

day3: PI3 ~ treatment + (1 | line)

Df AIC BIC logLik deviance Chisq Chi Df Pr(>Chisq)

day3nul 3 741.32 748.47 -367.66 735.32

day3 4 741.23 750.76 -366.61 733.23 2.092 1 0.1481

---

DAY 4

day4nul: PI4 ~ (1 | line)

day4: PI4 ~ treatment + (1 | line)

Df AIC BIC logLik deviance Chisq Chi Df Pr(>Chisq)

day4nul 3 723.60 730.75 -358.8 717.60

day4 4 720.41 729.93 -356.2 712.41 5.1955 1 0.02264 *

---

DAY 5

day5nul: PI5 ~ (1 | line)

day5: PI5 ~ treatment + (1 | line)

Df AIC BIC logLik deviance Chisq Chi Df Pr(>Chisq)

day5nul 3 747.34 754.49 -370.67 741.34

day5 4 743.24 752.77 -367.62 735.24 6.1034 1 0.01349 *

---
